# The Effects of Orally Administered Beta-Glucan on Innate Immune Responses in Humans, a Randomized Open-Label Intervention Pilot-Study

**DOI:** 10.1371/journal.pone.0108794

**Published:** 2014-09-30

**Authors:** Jenneke Leentjens, Jessica Quintin, Jelle Gerretsen, Matthijs Kox, Peter Pickkers, Mihai G. Netea

**Affiliations:** 1 Department of Internal Medicine, Radboud University Medical Center, Nijmegen, the Netherlands; 2 Department of Intensive Care Medicine, Radboud University Medical Center, Nijmegen, the Netherlands; 3 Radboud Institute for Infectious Diseases, Nijmegen, the Netherlands; Karolinska Institutet, Sweden

## Abstract

**Rationale:**

To prevent or combat infection, increasing the effectiveness of the immune response is highly desirable, especially in case of compromised immune system function. However, immunostimulatory therapies are scarce, expensive, and often have unwanted side-effects. β-glucans have been shown to exert immunostimulatory effects *in vitro* and *in vivo* in experimental animal models. Oral β-glucan is inexpensive and well-tolerated, and therefore may represent a promising immunostimulatory compound for human use.

**Methods:**

We performed a randomized open-label intervention pilot-study in 15 healthy male volunteers. Subjects were randomized to either the β -glucan (n = 10) or the control group (n = 5). Subjects in the β-glucan group ingested β-glucan 1000 mg once daily for 7 days. Blood was sampled at various time-points to determine β-glucan serum levels, perform *ex vivo* stimulation of leukocytes, and analyze microbicidal activity.

**Results:**

β-glucan was barely detectable in serum of volunteers at all time-points. Furthermore, neither cytokine production nor microbicidal activity of leukocytes were affected by orally administered β-glucan.

**Conclusion:**

The present study does not support the use of oral β-glucan to enhance innate immune responses in humans.

**Trial Registration:**

ClinicalTrials.gov NCT01727895

## Introduction

Defense mechanisms against invading pathogens are of vital importance to our survival. Therefore, to prevent or combat infection, increasing the effectiveness of the immune response is highly desirable. However, immunostimulatory therapies are scarce, expensive, and often have unwanted side-effects [1].

“Medicinal” mushrooms are used in alternative medicine throughout the world for their presumed enhancing effect on the immune system [2], [3]. Although a number of fungal components have been implicated in these properties, β-glucans (naturally occurring carbohydrates) have attracted the most attention [4]. Since the early 1900s, numerous *in vitro* and animal studies have demonstrated immunostimulatory effects of β-glucans [5]. In addition, the advent of molecular immunology has provided rigorous mechanistic explanations for how humans recognize glucans and how this may influence the immune system [6]. β-glucan is already applied as a food additive in animal feed to enhance the immune response [7] and it is also widely offered on the internet as a dietary supplement for humans, advertised to have beneficial immunostimulatory effects. Due to the fact that it is inexpensive and well tolerated, oral β-glucan appears as a promising candidate to enhance the immune response. However, there are no studies to substantiate the putative immunostimulatory effects of orally administered β-glucan in humans. The only evidence of immunological effects of oral β-glucan in humans to date is derived from a study in patients with advanced breast cancer, in which oral β-glucans enhanced expression of surface molecules associated with macrophage proliferation and activation in peripheral blood mononuclear cells (PBMCs) [8].

In the present study we investigated the effects of a commercially available orally administered water-insoluble β-glucan on immune responses of *ex vivo*-stimulated leukocytes in healthy volunteers.

## Methods

The protocol for this trial and supporting CONSORT checklist are available as supporting information; see Checklist S1 and Protocol S1 in the online supplement.

### Subjects

**Figure 1:**
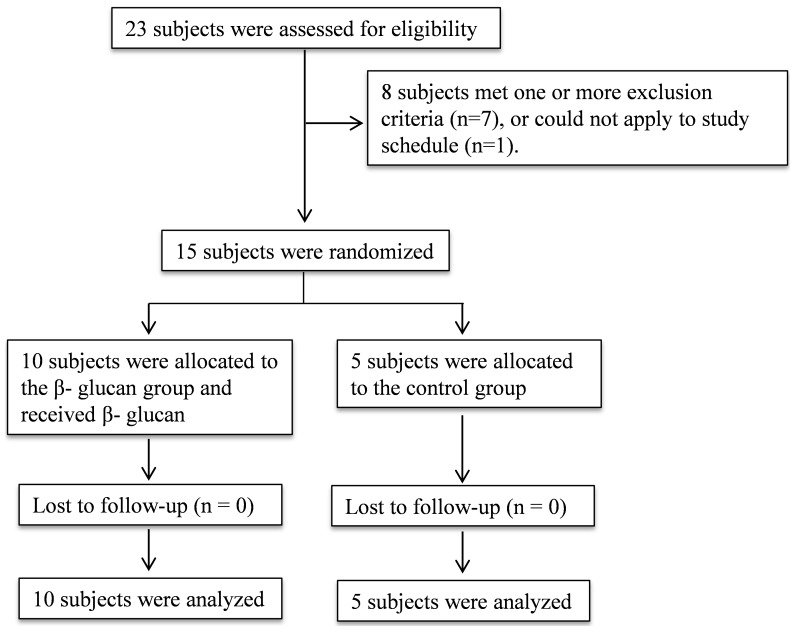
CONSORT flowchart.

This study was registered at ClinicalTrials.gov as NCT01727895. After approval from the local Ethics Committee of the Radboud University Nijmegen Medical Centre, 15 healthy male volunteers gave written informed consent to participate in the experiments which took place from May 2013 until July 2013 (Figure 1). All experiments were in accordance with the declaration of Helsinki. Subjects were screened before the start of the experiment. Subjects with febrile illness during the two weeks before the experiment were excluded, and subjects were not allowed to take any prescription drugs. Throughout the study period, subjects documented their consumption of β-glucan-containing foods in a “food-diary”, and the amount of consumed β-glucan-containing foods was limited in order to minimize the inter-individual variability.

### Study design

**Figure 2:**
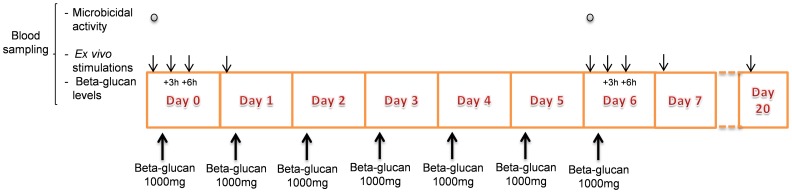
Experimental design of the study. The primary outcome measure was the TNF-α secretion by *ex vivo* lipopolysaccharide (LPS) -stimulated peripheral blood mononuclear cells (PBMC's). Secondary endpoints were the production of other cytokines (TNF-α, IL-6, IL-10, IL-1β, IL-17, IL-22, Interferon (IFN)-γ) by leukocytes *ex vivo* stimulated with various stimuli, β-glucan plasma levels, and Microbicidal activity of PBMC's.

We performed an open-label, intervention pilot-study in 15 healthy human volunteers. The study design is depicted in Figure 2. Briefly, subjects were randomized to either the β-glucan group (n = 10) or the control group (n = 5) using the sealed envelope method. Subjects in the β-glucan group ingested β-glucan (water-insoluble β-glucan derived from bakers yeast [*S. cerevisiae*] sold as a dietary supplement [Glucan #300, BG; Biothera, Eagan, Minnesota, USA, for Transferpoint, Columbia, USA, hereafter designated as BG]) 1000 mg once daily as recommended by the manufacturer, for 7 days. This preparation, has a purity of at least 83% guaranteed by the manufacturer. Before the first BG ingestion (on day 0) as well as 3, 6, and 24 hours afterwards, subjects came to the hospital for blood sampling and reporting of side-effects. This sampling schedule was repeated on day 6 (before and after the seventh BG ingestion). A final single sampling time-point took place on day 20. The subjects in the control group (n = 5) did not take BG, but the sampling schedule was otherwise identical.

### Cytokine measurements

Venous blood was drawn into EDTA tubes, after which peripheral blood mononuclear cells (PBMCs) were isolated as described previously [9]. In short, blood was diluted in phosphate buffered saline (PBS) (1∶1) and fractions were separated by Ficoll (Ficoll-Paque Plus, GE healthcare, Zeist, The Netherlands) density gradient centrifugation. Cells were washed twice with PBS and resuspended in RPMI-1640+ (RPMI-1640 Dutch modification supplemented with 10 µg/mL gentamicin, 10 mM L-glutamine, and 10 mM pyruvate) (Gibco, Invitrogen, Breda, The Netherlands). PBMCs were counted using a particle counter (Beckmann Coulter, Woerden, The Netherlands) and were plated in 96 well round-bottom plates (Corning, NY, USA) at a final concentration of 2.5×10^6^/mL, in a total volume of 200 µL. The PBMCs were stimulated for 24 hours, 48 hours, and 7 days with medium alone, or medium containing *E. coli* lipopolysaccharide (LPS; 10 ng/mL), heat-inactivated *Candida albicans* blastoconidia UC820 (1×10^6^ microorgansims/mL), Pam3Cys 10 µg/mL (EMC Microcollections), sonicated mycobacterium tuberculosis (MTB) H37Rv (1 µg/mL), poly(I:C) 50 µg/mL (Invivogen), S. *aureus* (1×10^7^ microorgansims/mL), antiCD3/antiCD28 2,5×10^5^ beads/well (Miltenyi Biotec). After stimulation, cell culture supernatant was collected and stored at −20°C. When all samples were collected, cytokines were measured using commercially available ELISAs (R&D Systems, MN, USA and Sanquin, Amsterdam, The Netherlands) according to the protocols supplied by the manufacturer.

### Microbicidal activity assay

Microbicidal activity assay was performed as previously described, using the fungal microorganism *Candida albicans* as a model pathogen [10]. Briefly, *C. albicans* UC820 yeast suspension was incubated with PBMCs isolated from the volunteers at day 0 (t = 0) and day 6 (t = 0) at a MOI of 1∶5 or 1∶50 in RPMI in a 96 wells plate. *C. albicans* UC820 suspension was obtained from an overnight culture in 25 ml liquid Sabouraud at 29°C. The *C. albicans* solution was washed three times with PBS, and the number of yeast cells counted in a hemacytometer. *C. albicans* were then incubated with PBMCs or no cells (control well) for 5 hours at 37°C. The amount of *C. albicans* added to the wells was determined by plating serial dilutions on Sabouraud plates in duplo. After a 5–hour incubation, the content of each well was recovered in sterile water and serially diluted. Serial dilutions were plated on Sabouraud plates in duplo. After 24 hours at 29°C, the CFU were counted. The candidacidal activity was calculated as a ratio of *Candida* growth with PBMCs vs. control wells (*Candida* alone, no cells).

### (1→3)-β-D-glucan detection in serum samples

β-glucan levels were determined for all subjects on several time points using the Fungitell kit (Associates of Cape Cod, Inc., Cape Cod, MA), according to the manufacturer's instructions [11]. Lower and upper detection limits were 31.25 and 500 pg/ml, respectively.

### Calculations and statistical analysis

Distribution of data was determined using Kolmogorov-Smirnov tests. Demographic data were analyzed using Mann-Whitney U tests. Comparisons between the two groups over time were made using repeated measures two-way analysis of variance (ANOVA, interaction term). *Ex vivo* stimulation cytokine data were log-transformed to obtain a normal distribution. A p-value of <0.05 was considered statistically significant. Calculations and statistical analyses were performed using Graphpad Prism version 5.0 (Graphpad Software, San Diego, CA, USA).

## Results

### Demographic characteristics

Demographic characteristics of the study population are listed in Table 1. There were no significant differences in baseline characteristics between the two study groups. No serious adverse events occurred during the trial. BG intake was well tolerated, with no side effects reported besides one subject who reported a flare of acne one week after discontinuation of BG for which he contacted his general practitioner.

**Table 1:** Demographic characteristics.

	β-glucan (n = 10)	Control (n = 5)	p-value
Age (years)	20 [19]–[22]	20 [19]–[24]	1.00
Hight (cm)	182 [179–188]	183 [177–196]	0.78
Weight (kg)	81 [74–87]	80 [74–87]	0.90
BMI (kg/m^2^)	24.1 [22.9–26.5]	23.9 [23.6–25.1]	0.95

BMI: body mass index. Data are presented as median and interquartile range. p-values calculated using Mann-Whitney U-tests.

### (1→3)-β-D-glucan serum levels

Following oral administration, β-glucan was barely detectable in serum of volunteers at all time-points, and this was not significantly different from the β-glucan levels observed in the placebo group (data not shown).

### Cytokine production by ex vivo-stimulated PBMCs

**Figure 3:**
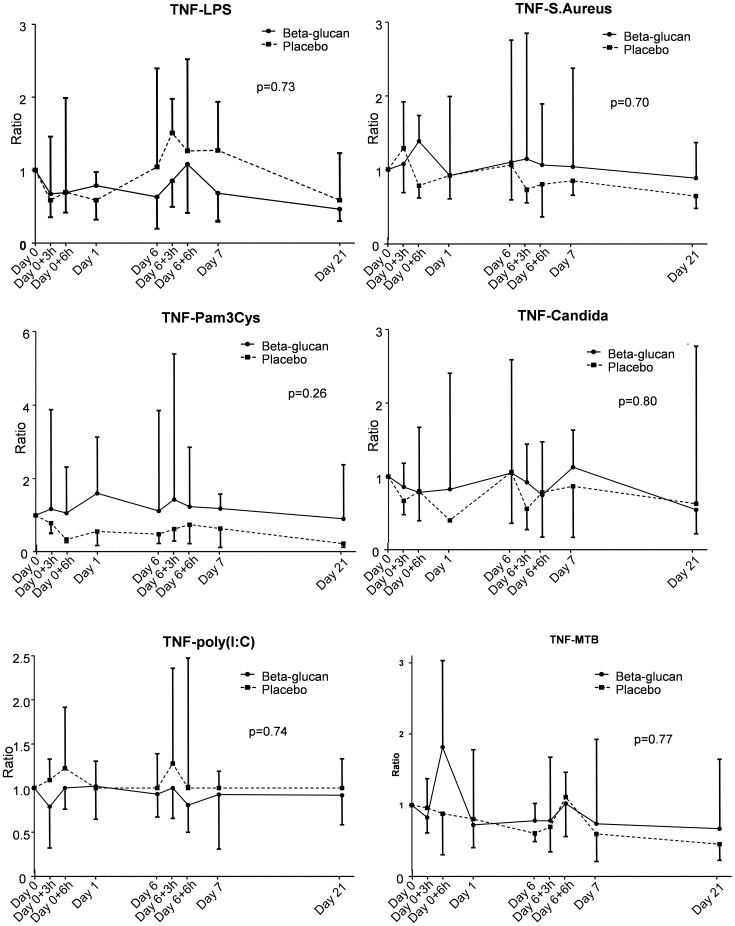
Effect of oral β-glucan on *ex vivo* TNF-α production by PBMCs stimulated for 24 hours with lipopolysaccharide (LPS), Pam3Cys, Poly(I:C), S. *aureus,* C. *albicans*, or M. *tuberculosis* (MTB). To correct for possible baseline differences between groups, concentrations at day 0 are set at 1, and concentrations at subsequent time-points are plotted as ratios (median and interquartile range). Baseline (day 0) TNF-α concentrations in pg/ml [interquartile range] of subjects in the β-glucan and control groups were: LPS: 691 [276–2649] and 749 [287–1160]), Pam3Cys: 855 [590–1727] and 1242 [622–3567], Poly(I:C): 107 [78–399] and 125 [78–250], S. *aureus:* 21948 [14347–41751] and 19424 [12140–31722], C. *albicans:* 10875 [5860–14159] and 6208 [3924–14943], and MTB: 424 [285–1980] and 1153 [218–2461]. P values between groups were calculated using repeated measures two-way analysis of variance (ANOVA, interaction term) on log transformed data.

The production of the archetypal proinflammatory cytokine tumor necrosis factor (TNF)-α after incubation with various stimuli at different time points showed considerable interindividual variation, but it was not affected by β-glucan intake (Figure 3). Furthermore, no relevant differences in TNF-α production compared with the control group were observed. Likewise, no effects of BG intake within the β-glucan group or between the β-glucan group and the control group were observed with regard to the production of interleukin (IL)-6, IL-1β, IL-10, Interferon (IFN)-γ, IL-17 and IL-22 (Figure S1, Panel A–D, in the online supplement).

### Microbicidal activity

**Figure 4:**
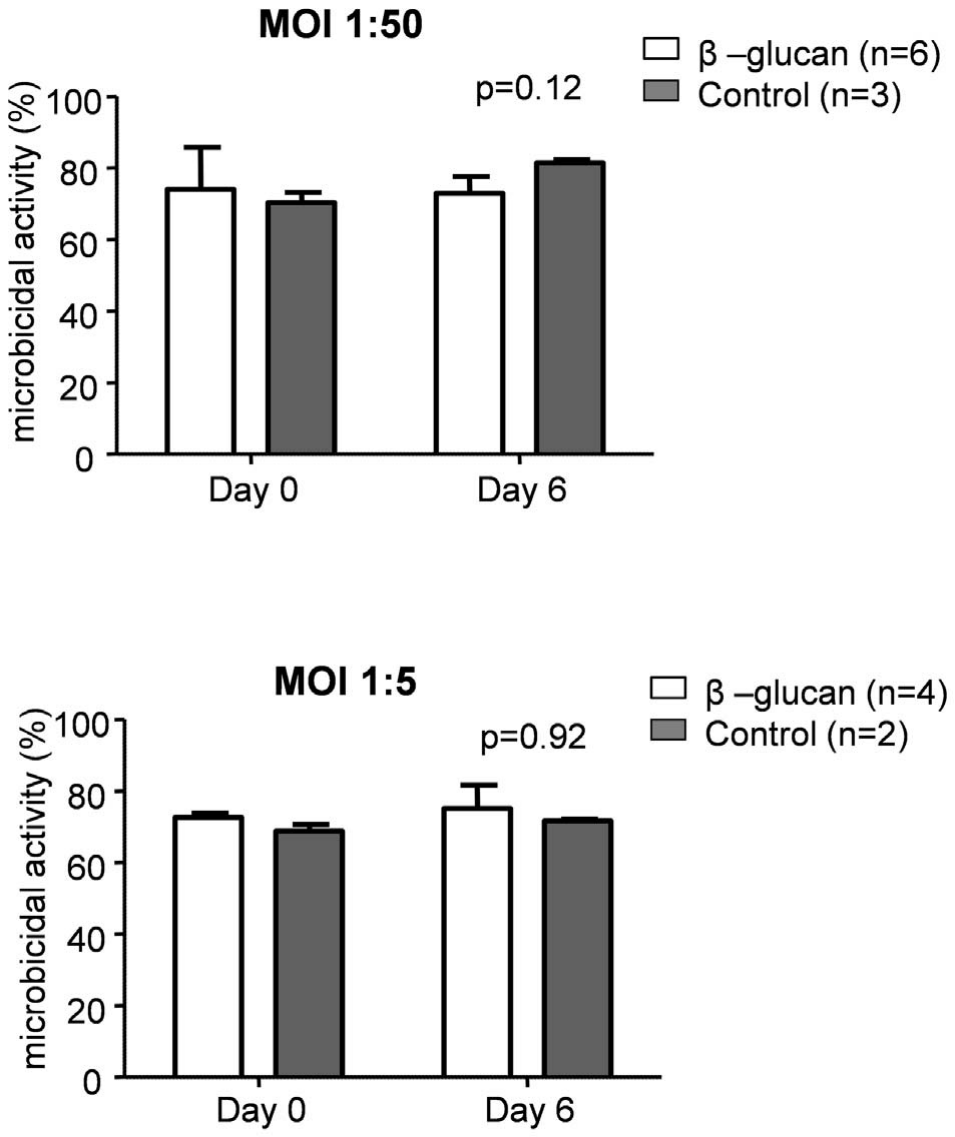
Microbicidal activity of PBMCs isolated from the volunteers at day 0 (before β-glucans intake), or at day 6 (after six days of β-glucan intake) from β-glucan (white) or from control (grey) volunteers. The microbicidal activity was calculated as a ratio of *Candida* growth with PBMCs vs. control wells (*Candida* alone, no cells). Data are presented as mean ± SEM. P values between groups were calculated using repeated measures two-way analysis of variance (ANOVA, interaction term).

We assessed the candidacidal properties of PBMCs isolated from volunteers before and 6 days after the administration of β-glucan, and at the same time-points in the control group. No significant differences could be observed in candidacidal activity in both groups, using multiplicity of infection (MOI) of either 1∶50 or 1∶5 (Figure 4).

## Discussion

In the present study, we show that daily intake of 1000 mg commercially available oral β-glucan for 7 days does not result in detectable serum β-glucan levels in healthy human volunteers, and that it does not modulate the innate immune response, as reflected by leukocyte-derived cytokine production upon *ex vivo* stimulation and microbicidal activity.

Because of increasing evidence for immunomodulatory properties of β-glucans [5], [10], and because commercially available oral β-glucans are widely advertised as having beneficial immunostimulatory effects, we wanted to assess their biological effects in humans. β-glucans are glucose polymers which can be isolated from cell walls of yeasts and mushrooms, but also from cell walls of other fungi, bacteria, algae, and cereal grains [5]. Their activity has been shown to be influenced by their degree of branching, size, molecular structure [12], [13], and the purity of the administered β-glucan [12]. The most active β-glucans reportedly share a common structure: a main chain consisting of (l-3)-linked β-D glucopyranosyl units, along which are randomly dispersed single β-D-glucopyranosyl units attached by l-6 or 1-4 linkages [5]. The BG used in this study is a polymer of β-(1-3)-D-glycopyranosyl units with branching at β-(1-6)-D-glycopyranosyl units, therefore belonging to the class of most active β-glucans. In addition, in an evaluation of four different orally administered commercially available β-glucans in mice, BG was shown to be the most potent in increasing phagocytosis capacity of peripheral blood cells and IL-2 production of splenic cells [10]. Based on these data, and because BG is rated ‘GRAS’ (Generally Recognized As Safe) by the Food and Drug Administration (FDA) as a dietary food additive supplement [14], and safe and non-toxic by the Dutch Medicines Evaluation Board (MEB) [15], we deemed this preparation of BG representative of commercially available oral β-glucans. Because of the complete absence of any effect on cytokine production or microbicidal activity, although investigated in a small number of subjects for only a limited period, we conclude that BG or similar glucan products do not appear to be viable approaches for immunostimulation in humans.

The lack of immunostimulatory effects of β-glucan in our study could have several reasons. First, it could be due to the absence of adequate absorption of β-glucans from the intestinal tract in healthy volunteers, which is in agreement with the undetectable serum levels found in various other studies showing that enteral administration of insoluble β-glucans from different sources result in low systemic blood levels (less than 0.5%) in mice [16], and weaned pigs [17]. Nevertheless, despite these low systemic blood levels, significant systemic immunomodulating effects in terms of humoral and cellular immune responses were demonstrated. It was speculated that these effects are exerted by enterocytes that facilitate the transportation of β-glucans and similar compounds across the intestinal cell wall into the lymph fluid, where they interact with macrophages and thereby activate the immune system despite low serum levels [18]. Given the lack of effects of BG in our study, it is unlikely that these local effects also take place to a significant degree in humans.

A second explanation might be related to the water solubility of β-glucans, because previous studies have demonstrated that water soluble β-glucans interact with the immune system differently compared with their insoluble counterparts. Soluble β-glucans activate the immune system in a complement mediated manner, depending on specific antibodies[19], while insoluble β-glucans, like BG used in this study, activate both the innate and the adaptive immune responses via the dectin-1 receptor pathway [19], [20]. As such, it has been suggested that soluble β-glucans possess less biological activity than their insoluble counterparts. Therefore, although we did not investigate a soluble formula, it is unlikely that orally administered water soluble β-glucans will have a more pronounced immunostimulatory effect in humans than the BG used in this study. However, future studies are warranted to assess this aspect.

Third, the lack of effects might be related to the fact we used a commercially available β-glucan preparation sold as a dietary supplement, with a relatively modest purity of at least 83%. It is possible that a highly purified pharmaceutical preparation optimized for oral delivery could have immunostimulatory effects in humans. Finally, we only investigated one dose of BG, based on the recommendation of the manufacturer, which is not supported by empirical data. Nevertheless, this dose is similar to that used in two previous studies, where it was demonstrated that oral ingestion of 900 mg water-insoluble β-glucan daily for 16 or 26 weeks reduced the incidence in common cold episodes during the cold season [21], [22]. Of note, endpoints in both studies were based on questionaires, no immunological endpoints were assessed. Also, the dose used in the present study is higher than the dose rated ‘GRAS’ by the FDA and MEB (375 mg per day) [14] [15], which was based on the mean background intake of β-glucans from other dietary sources.

While our study does not support use of oral β-glucan to enhance the immune response, it does not exclude that parental administration of β-glucan, as currently performed in clinical trials (e.g. NCT01269385, NCT00002099), could represent a valuable immunostimulatory therapy. It has been reported that β-glucan from S. *cerevisiae* administered intravenously to high-risk surgical patients resulted in decreased infection incidence, reduced need for antibiotics, shortened intensive care unit length stay, and ultimately improved survival compared with placebo [23]. In addition, several clinical trials in East Asia have proposed immunomodulatory and antioncogenic effects of β-glucan in cancer patients [24]–[26]. Larger studies are warranted to confirm these data, investigate the biological mechanisms though which effects are induced, and to fully explore the therapeutic potential of β-glucans.

## Conclusion

Despite promising results obtained in *in vitro* and animal studies, our study demonstrates that the use of oral β-glucan does not enhance the responsiveness of the immune system in humans. Future efforts should concentrate on assessing the immunological and clinical effects of intravenous β-glucan preparations.

## Supporting Information

Figure S1 **Effect of oral β-glucan on** ***ex vivo*** **IL-6, IL-1β, IL-10, IFN-γ, IL-17, and IL-22 production by PBMCs stimulated for 24 or 48 hours with different stimuli.**
(DOC)

Checklist S1 **CONSORT checklist.**
(DOC)

Protocol S1 **Protocol of this trial.**
(PDF)
