# Supplementary material for: The Effects of Orally Administered Beta-Glucan on Innate Immune Responses in Humans, a Randomized Open-Label Intervention Pilot-Study
Source: PLoS One. 2014 Sep 30;9(9):e108794. doi: 10.1371/journal.pone.0108794 (PMC4182605; doi:10.1371/journal.pone.0108794)
Supplement: Figure S1 — Effect of oral β-glucan on ex vivo IL-6, IL-1β, IL-10, IFN-γ, IL-17, and IL-22 production by PBMCs stimulated for 24 or 48 hours with different stimuli. (DOC) [file pone.0108794.s001.doc]

**ONLINE SUPPLEMENT**

**
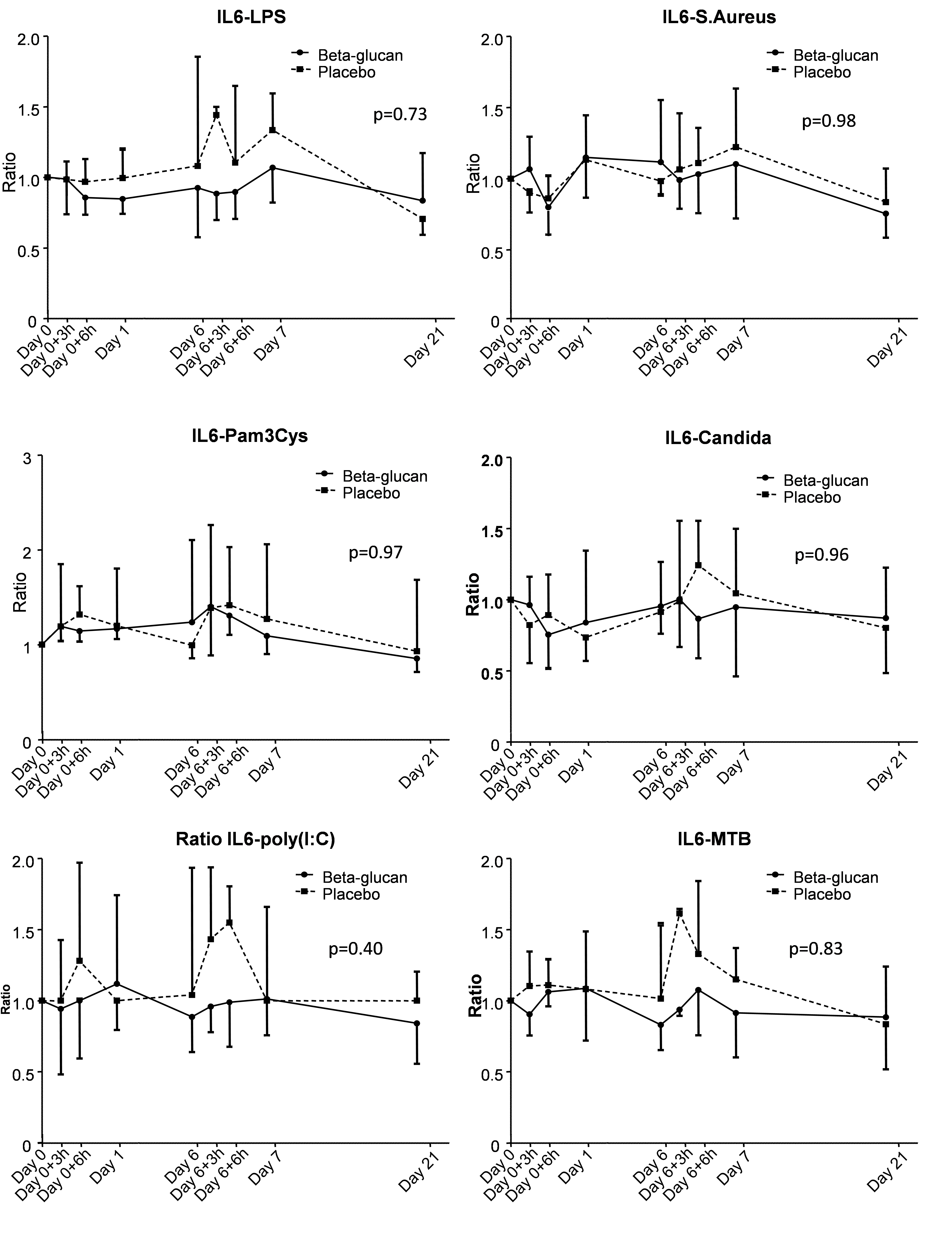
Supplementary Figure 1A:** Effect of oral β-glucan on *ex vivo* IL-6 production by PBMCs stimulated for 24 hours with lipopolysaccharide (LPS), Pam3Cys, Poly(I:C), S. a*ureus,* C. *albicans*, or M. *tuberculosis* (MTB). To correct for possible baseline differences between groups, concentrations at day 0 are set at 1, and concentrations at subsequent time-points are plotted as ratios (median and interquartile range). Baseline (day 0) IL-6 concentrations in pg/ml [interquartile range] of subjects in the β-glucan and control groups were: LPS: 22862 [37588-11284] and 14516 [13980-17357], Pam3Cys: 33293 [15632-40949] and 12258 [9957-22041], Poly(I:C): 5308 [1618-8228] and 937 [468-2964], S. *Aureus*: 19277 [12123-24092] and 11585 [8093-15266] *,* C. *Albicans*: 11130 [8899-15794] and 8924 [5168-10147], and MTB: 21997 [10416-35469] and 12470 [12232-15365]. P values between groups were calculated using repeated measures two-way analysis of variance (ANOVA, interaction term) on log transformed data.

**Supplementary Figure 1B:** Effect of oral β-glucan on *ex vivo* IL-β production by PBMCs stimulated for 24 hours with lipopolysaccharide (LPS), Pam3Cys, Poly(I:C), S. *aureus,* C. *albicans*, or M. *tuberculosis* (MTB). To correct for possible baseline differences between groups, concentrations at day 0 are set at 1, and concentrations at subsequent time-points are plotted as ratios (median and interquartile range). Baseline (day 0) IL-β concentrations in pg/ml [interquartile range] of subjects in the β-glucan and control groups were: LPS: 5600 [2604-12624] and 4061 [2322-10436], Pam3Cys: 9295 [3688-11162] and 5189 [3303-6686], Poly(I:C): 233 [39-412] and 142 [39-266], S. *Aureus*: 24141 [20536-29528] and 15456 [14697-24582]*,* C. *Albicans:* 6975 [5327-8954] and 6187 [3832-8224], and MTB: 2574 [1680-7120] and 3313 [1211-6465]. P values between groups were calculated using repeated measures two-way analysis of variance (ANOVA, interaction term) on log transformed data.


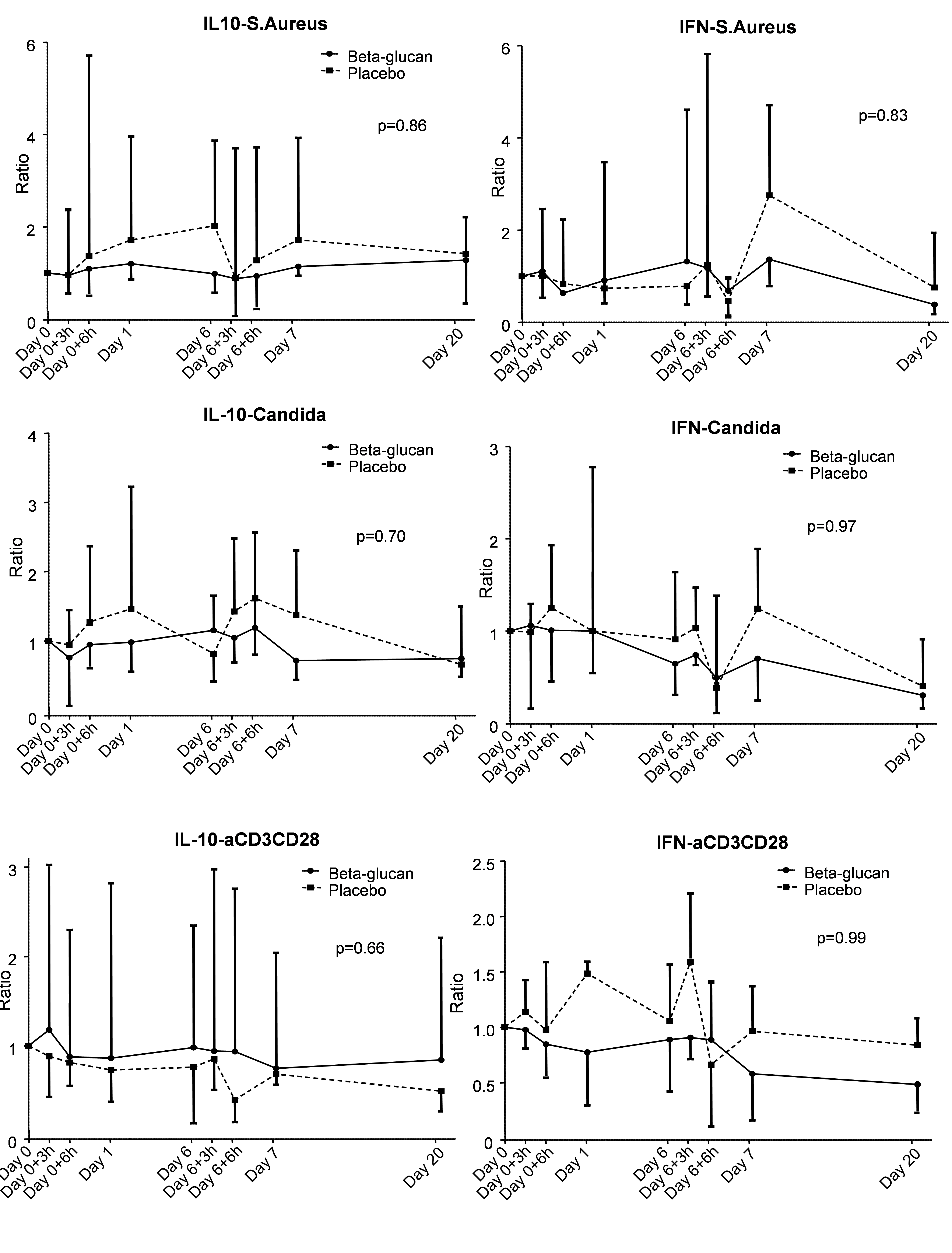


**Supplementary Figure 1C:** Effect of oral β-glucan on *ex vivo* Interleukin-10 and Interferon-gamma (IFN) production by PBMCs stimulated for 48 hours with S. *aureus,* C. *albicans*, or anti-CD3CD28. To correct for possible baseline differences between groups, concentrations at day 0 are set at 1, and concentrations at subsequent time-points are plotted as ratios (median and interquartile range). Baseline (day 0) IFN concentrations in pg/ml [interquartile range] of subjects in the β-glucan and control groups were: S. *Aureus:*24950 [10491-48275] and 21100 [5633-37350]*,* C. *Albicans:* 22100 [13450-37225] and 26400 [14563-40550], and anti-CD3CD28: 35900 [12628-50000] and 27300 [15800-65075]. Baseline (day 0) IL-10 concentrations in pg/ml [interquartile range] of subjects in the β-glucan and control groups were: S. *Aureus:* 405 [158-503] and 140 [59-267]*,* C. *Albicans:* 83 [44-189] and 39 [25-93], and anti-CD3CD28: 160 [102-229] and 129 [77-362]. P values between groups were calculated using repeated measures two-way analysis of variance (ANOVA, interaction term) on log transformed data.

**
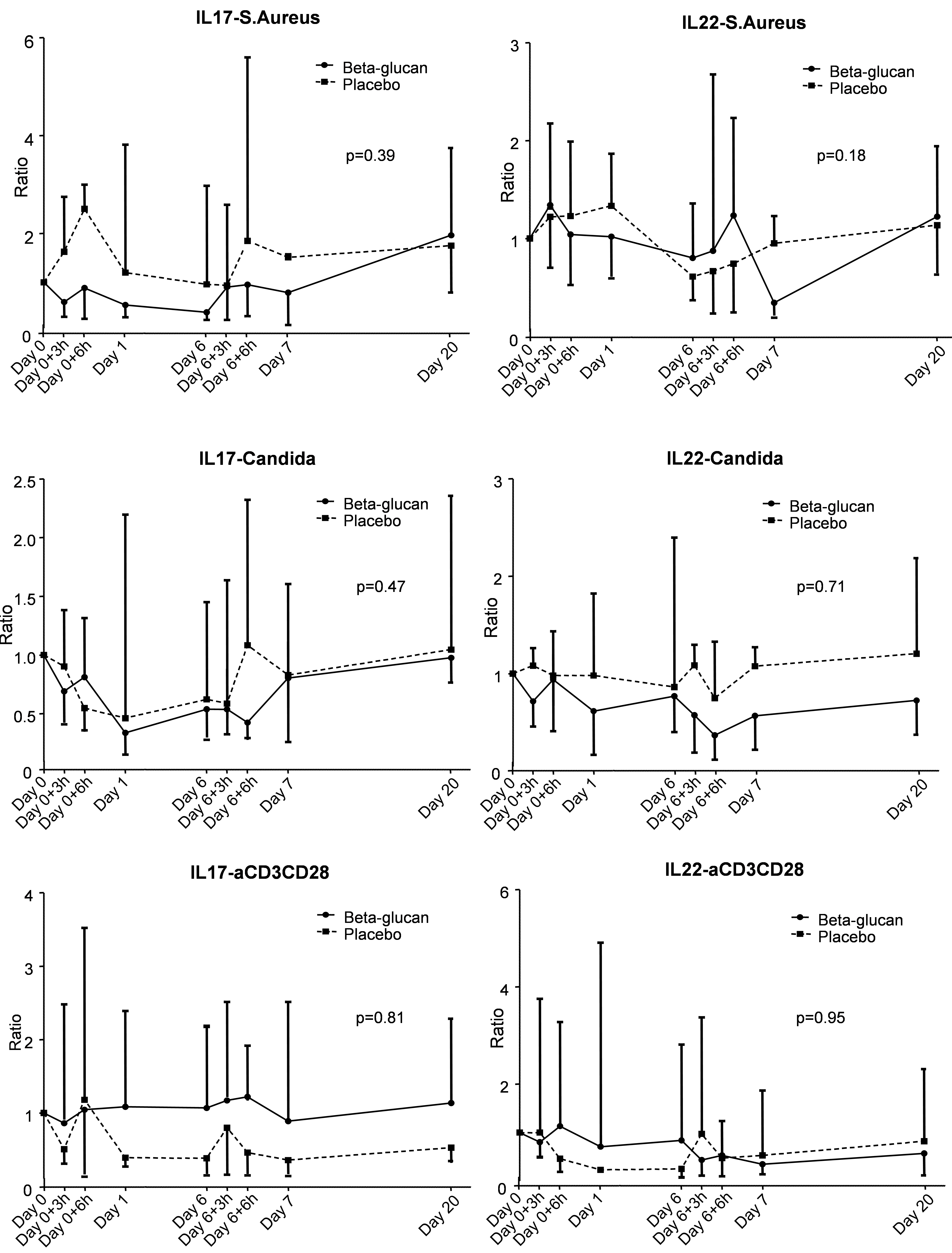
**

**Supplementary Figure 1D:** Effect of oral β-glucan on *ex vivo* Interleukin-17 and -22 production by PBMCs stimulated for 7 days with S. *aureus,* C. *albicans*, or anti-CD3CD28. To correct for possible baseline differences between groups, concentrations at day 0 are set at 1, and concentrations at subsequent time-points are plotted as ratios (median and interquartile range). Baseline (day 0) IL-10 concentrations in pg/ml [interquartile range] of subjects in the β-glucan and control groups were: S. *Aureus:* 245 [116-470] and 200 [70-563]*,* C. *Albicans:* 885 [606-1621] and 940 [720-1365], and anti-CD3CD28: 488 [219-580] and 360 [235-1268]. Baseline (day 0) IL-22 concentrations in pg/ml [interquartile range] of subjects in the β-glucan and control groups were: S. *Aureus:* 2620 [1089-12670] and 6145 [2140-9165]*,* C. *Albicans:* 2730 [1694-9335] and 6875 [5588-10440], and anti-CD3CD28: 250 [147-1623] and 1175 [363-1225]. P values between groups were calculated using repeated measures two-way analysis of variance (ANOVA, interaction term) on log transformed data.
