# Supplementary material for: The Effects of Orally Administered Beta-Glucan on Innate Immune Responses in Humans, a Randomized Open-Label Intervention Pilot-Study
Source: PLoS One. 2014 Sep 30;9(9):e108794. doi: 10.1371/journal.pone.0108794 (PMC4182605; doi:10.1371/journal.pone.0108794)

RESEARCH PROTOCOL
Version 3

Effects of orally administered Beta-glucan on leukocyte function in humans, a pilot study

(March 2013)

PROTOCOL TITLE

Effects of orally administered Beta-glucan on leukocyte function in humans, a pilot study

**Protocol ID:**	Betaglucan_immunity
**Short title:**	Effects of oral Beta-Glucan on human leukocytes
**Version :**	Version 2
**Date :**	November 2012
**Coordinating investigator/project leader:**	Prof. Dr. M. Netea, internist, infectioloog<br>Radboud University Nijmegen Medical Centre<br>Department of Medicine, Nijmegen Institute for Infection, Inflammation and Immunity (N4i), PO box 9101, 6500 HB Nijmegen, The Netherlands
**Principal investigator:**	Drs. J. Leentjens, Internist in opleiding<br>Radboud University Nijmegen Medical Centre<br>Departments of Internal and Intensive Care Medicine, PO box 9101, 6525 GA Nijmegen, The Netherlands
**Associate investigators:**	M. Kox, MSc, PhD<br>Radboud University Nijmegen Medical Centre<br>Department of Intensive Care Medicine, PO box 9101, 6525 GA Nijmegen, The Netherlands<br>J. Quintin, PhD<br>Radboud University Nijmegen Medical Centre<br>Department of Medicine, Nijmegen Institute for Infection, Inflammation and Immunity (N4i), PO box 9101, 6500 HB Nijmegen, The Netherlands<br>Prof. Dr. P. Pickkers, Internist-intensivist<br>Radboud University Nijmegen Medical Centre<br>Department of Intensive Care Medicine, PO box 9101, 6525 GA Nijmegen, The Netherlands<br>Prof. Dr. J.G van der Hoeven, Internist-intensivist<br>Radboud University Nijmegen Medical Centre<br>Department of Intensive Care Medicine, PO box 9101, 6525 GA Nijmegen, The Netherlands
**Sponsor:**	Vici grant of the Netherlands Organization for Scientific Research (to M.G.N.)
**Independent physician(s):**	Drs. A. Rennings, internist<br>Radboud University Nijmegen Medical Centre<br>Department of Internal Medicine<br>6525 GA Nijmegen, The Netherlands
**Laboratory sites:**	Department of Experimental Internal Medicine<br>Radboud University Nijmegen Medical Centre<br>Postbus 9101, 6500 HB Nijmegen, the Netherlands

PROTOCOL SIGNATURE SHEET

**Name**	**Signature**	**Date**
Coordinating Investigator/Project leader:<br><br>Prof. M.G. Netea, Internist-infectioloog		30-10-2012
Principal Investigator:<br><br>Drs. J. Leentjens, internist in opleiding		30-10-2012

LIST OF ABBREVIATIONS AND RELEVANT DEFINITIONS

ABR	ABR form (General Assessment and Registration form) is the application form that is required for submission to the accredited Ethics Committee (ABR = Algemene Beoordeling en Registratie)
AE	Adverse Event
AR	Adverse Reaction
CA	Competent Authority
CO	Carbon monoxide
CCMO	Central Committee on Research Involving Human Subjects
CRP	C reactive protein
CV	Curriculum Vitae
DSMB	Data Safety Monitoring Board
etCO	End tidal carbon monoxide
EU	European Union
EudraCT	European drug regulatory affairs Clinical Trials GCP Good Clinical Practice
GM-CSF	Granulocyte macrophage colony stimulating factor
HMGB-1	High mobility group box 1
IB	Investigator's Brochure
IC	Informed Consent
ICAM	Inter-Cellular Adhesion Molecule
IMP	Investigational Medicinal Product
IMPD	Investigational Medicinal Product Dossier
IL-	Interleukin-
IFN-γ	Interferon gamma
LPS	Lipopolysaccharide
MAP	Mean arterial blood pressure
MCP	Monocyte chemoattractive protein
MODS	Multiple organ dysfunction syndrome
METC	Medical research ethics committee (MREC); in Dutch: medisch ethische toetsing commissie (METC)
NF-kB	Nuclear factor-kB
PBMC	Peripheral Blood Mononuclear Cell
UGT1A1	UPD glucuronosyl transferase enzyme
R(N)(O)S	Reactive (nitrogen) oxygen species
SAE	Serious Adverse Event
SPC	Summary of Product Characteristics (in Dutch: officiële productinformatie IB1-tekst)
Sponsor	The sponsor is the party that commissions the organisation or performance of the research, for example a pharmaceutical company, academic hospital, scientific organisation or investigator. A party that provides funding for a study but does not commission it is not regarded as the sponsor, but referred to as a subsidising party.
SUSAR	Suspected Unexpected Serious Adverse Reaction
TNF	Tumor necrosis factor
VCAM	Vascular cell adhesion molecule
VWF	Von Willebrand Factor
Wbp	Personal Data Protection Act (in Dutch: Wet Bescherming Persoonsgevens)
WMO	Medical Research Involving Human Subjects Act (Wet Medisch-wetenschappelijk Onderzoek met Mensen)

1 INTRODUCTION AND RATIONALE ... 9
2 OBJECTIVES ... 12
3 STUDY DESIGN ... 13
4 STUDY POPULATION ... 14
4.1 Population (base): ... 14
4.2 Inclusion criteria ... 14
4.3 Exclusion criteria ... 14
4.4 Sample size calculation ... 14
5 TREATMENT OF SUBJECTS ... 15
5.1 Investigational product/treatment ... 15
5.2 Use of co-intervention ... 15
5.3 Escape medication ... 15
6 INVESTIGATIONAL MEDICINAL PRODUCT ... 16
6.1 Name and description of investigational medicinal product ... 16
6.2 Summary of findings from non-clinical studies ... 16
6.3 Summary of findings from clinical studies ... 16
6.4 Summary of known and potential risks and benefits ... 16
6.5 Description and justification of route of administration and dosage ... 16
6.6 Dosages, dosage modifications and method of administration ... 16
6.7 Preparation and labeling of Investigational Medicinal Product ... 16
6.8 Drug accountability ... 16
7 METHODS ... 17
7.1 Study parameters/endpoints ... 17
7.1.1 Main study parameter/endpoints ... 17
7.1.2 Secondary study parameters/endpoints ... 17
7.1.3 Other study parameters ... 17
7.2 Randomisation, blinding and treatment allocation ... 17
7.3 Study procedures ... 17
7.3.1 Time course ... 18
7.3.2 Laboratory tests ... 19
7.3.3 Methods of determination ... 20
7.4 Withdrawal of individual subjects ... 20
7.4.1 Specific criteria for withdrawal ... 20
7.5 Replacement of individual subjects after withdrawal ... 21
7.6 Follow-up of subjects withdrawn from treatment ... 21
7.7 Premature termination of the study ... 21
SAFETY REPORTING ... 22
7.8 Section 10 WMO event ... 22
7.9 Adverse and serious adverse events ... 22
7.9.1 Adverse event ... 22
7.9.2 Serious Adverse Events ... 22
7.9.3 Suspected unexpected serious adverse reactions (SUSAR) ... 22

7.10 Follow-up of adverse events ... 23
7.11 Data Safety Monitoring Board (DSMB) ... 23
7.12 Abnormal laboratory tests results ... 23
8 STATISTICAL ANALYSIS ... 24
8.1 Modified intention to treat analysis ... 24
8.2 Descriptive statistics ... 24
8.3 Multivariate analysis ... 24
8.4 Interim analysis ... 24
9 ETHICAL CONSIDERATIONS ... 25
9.1 Regulation statement ... 25
9.2 Recruitment and consent ... 25
9.3 Benefits and risks assessment, group relatedness ... 25
9.4 Compensation for injury ... 25
9.5 Incentives ... 26
10. ADMINISTRATIVE ASPECTS, MONITORING AND PUBLICATION ... 27
10.1 Handling and storage of data and documents ... 27
10.2 Monitoring and Quality Assurance ... 27
10.3 Amendments ... 27
10.4 Annual progress report ... 27
10.5 End of study report ... 27
10.6 Public disclosure and publication policy ... 28
11. STRUCTURED RISK ANALYSIS ... 29
11.1 Potential issues of concern ... 29
12.2 Synthesis ... 29
12 REFERENCES ... 30

Appendix 1.

STUDY SYNOPSIS

Title Effects of orally administered Beta-glucan on leukocyte function in humans, a pilot study

Indication Immune stimulatory therapies

Objectives *Primary objective:*
The primary objective of the study is to evaluate the effects of orally administered Beta-glucan on human leukocyte function. The effects of Beta-glucan will be determined by measuring the *ex vivo* responsiveness of leukocytes to various inflammatory stimuli, as well as phagocytosis and killing of pathogens, as markers of the antimicrobial response, and the pathogen phagocytosis and killing capacity of leukocytes. The primary outcome measure is the TNF-α secretion by *ex vivo* LPS-stimulated peripheral blood mononuclear cells (PBMCs).

Secondary Objective(s): There are 6 secondary objectives:

1. To determine the production of other cytokines (TNF-α, IL-6, IL-10, IL-1β, IL-17, IL-22, Interferon (IFN)-γ) by leukocytes *ex vivo* stimulated with various stimuli (including LPS, Pam3Cys, Mycobacterium tuberculosis, Poly(I:C), Candida albicans, staphylococcus aureus).
2. To determine the absorbance of orally administered Beta-glucan into the blood compartment, measured by ELISA.
3. To determine the effects of Beta-glucan on changes in phenotype and gene expression caused by mechanisms other than changes in the underlying DNA sequence (epigenetic modifications).
4. To determine the effects of Beta-glucan on transcriptional pathways (by use of microarrays) with focus on inflammatory pathways.
5. To determine the effects of Beta-glucan on the leukocyte capacity to phagocytose and kill the fungal pathogen *Candida albicans*.
6. To determine the effects of Beta-glucan on faecal microbiota

Study design An open-label, intervention pilot-study in healthy human volunteers.

Sample size 15 healthy volunteers.

Eligibility criteria *Inclusion criteria*

- Written informed consent
- Age ≥18
- Healthy

Exclusion criteria

- Subjects with a history of allergy or intolerance to Beta-glucan
- Use of any medication
- Participation in a drug trial or donation of blood 3 months prior to Beta-glucan administration
- Use of antibiotics, norit, laxatives (up till 6 months prior to inclusion), cholestyramine, acid burn inhibitors or immune

suppressive agents (up till 3 months prior to inclusion), and pre- and probiotics (up till 1 month prior to inclusion).

Drugs and dosage Commercial available Beta-glucan derived from bakers yeast (S. Cerevisiae): Glucan #300® produced by Transferpoint, Columbia, United States. 2 capsules of 500mg Glucan #300®, daily, for seven days.

Route of administration Oral administration.

Procedures 10 Healthy volunteers will take Beta-glucan 1000 mg daily for seven days, 5 other healthy volunteers will serve as a control group and do not have to take Beta-glucan. The *ex-vivo* responsiveness of leukocytes to various inflammatory stimuli, peripheral blood mRNA expression of genes related to the immune response, epigenetic modifications, the leukocyte capacity to phagocytose and kill the fungal pathogen *Candida albicans*, blood levels of Beta-glucan, and faecal microbiota will be analyzed on different timepoints.

Statistical analysis Descriptive statistics.

1 INTRODUCTION AND RATIONALE

For centuries mankind has tried to improve their defense mechanisms against invading pathogens. The innate immunity is the first line of defense after the body's mechanical barriers. In many pathological situations that occur naturally (for example sepsis) or iatrogenic (for example chemotherapy), enhancement of the immune response is desirable. However, immune stimulating therapies are scarce, expensive, and often have undesirable side-effects.
The immunostimulatory properties of mushrooms have been recognized for centuries, and "medicinal" mushrooms are still widely used in alternative medicine all over the world. Although a number of fungal components have been implicated in these properties, Beta-glucans have attracted the most attention [1]. Numerous *in vitro* and animal studies have demonstrated immunomodulatory and anticancer effects of Beta-glucans. Moreover, recent molecular immunological studies have elucidated the immunological mechanisms behind recognition of glucans by immune cells and subsequent alterations of cellular responses [2]. However, although Beta-glucans are widely used as a health food supplement, their immunomodulatory effects after administration in humans have not yet been determined.

Structure
Structurally, all Beta-glucans are glucose polymers linked together by a 1-3 linear Beta-glycosidic chain core that differ from each other by their length and branching configuration [2, 3]. The branches derived from the glycosidic chain core are either 1-4 or 1-6 glycosidic chains that appear to be dependent on the source. Beta-glucans can not only be isolated from cell walls of mushrooms and yeasts, but also from cell walls of other fungi, bacteria, algae, and cereal grains [4]. Their activity is influenced by their degree of branching, size, and their molecular structure [5, 6], but also by the purity of the preparation [6]. The most active Beta-glucans have a common structure: a main chain consisting of (l-3)-linked Beta-D glucopyranosyl units along which are randomly dispersed single Beta-D-glucopyranosyl units attached by l-6 or 1-4 linkages [4] (Figure 1).

Immunity
Beta-glucans are not found in animals and are considered to be classic pathogen-associated molecular patterns (PAMPs) [7] which are recognized by the innate immune system of vertebrates, as well as invertebrates. The ability of the immune system to recognize and respond to PAMPs is dependent on pattern recognition receptors (PRRs) that have been identified on various human immune cells including monocytes, macrophages, neutrophils, and NK cells. To date, there have been four different Beta-glucan PRRs described including complement receptor 3 (CR3) [8], scavenger receptors [9], lactosylceramide [10], and most recently dectin 1 [11]. By binding to these PRRs, Beta-glucans are potent activators of the innate immune system and they are most well known for their ability to stimulate the immune system and boost resistance to various viral, bacterial, protozoan, and fungal diseases, as well as to promote antitumor activity. However, up till now, these effects have only been established *in vitro* and in animal models.

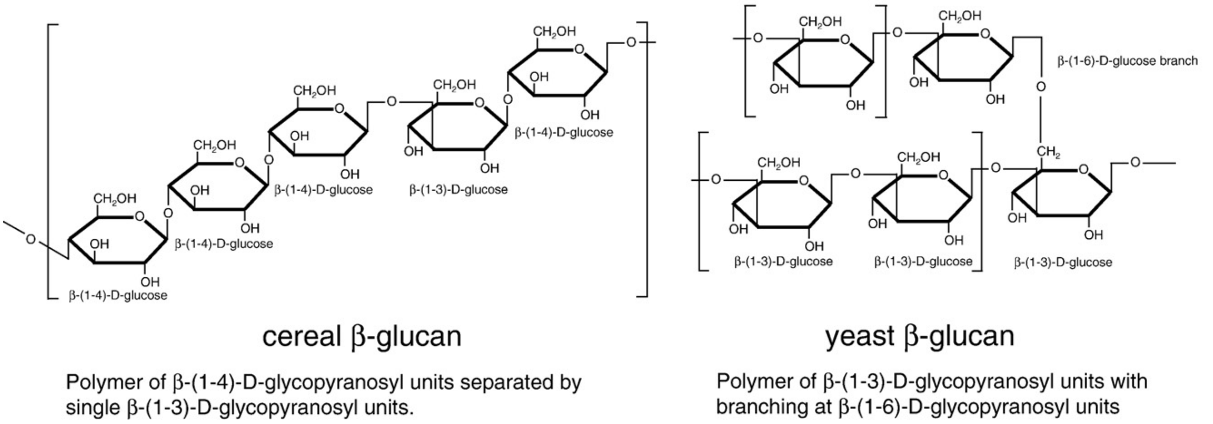

β-Glucan type	Structure	Description
Bacterial		Linear β1,3 glucan (i.e. Curdlan)
Fungal	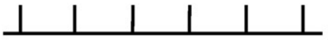	Short β1,6 branched, β1,3 glucan (i.e. Schizophyllan)
Yeast	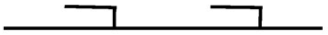	Long β1,6 branched, β1,3-glucan (i.e. WGP β-glucan, Betafectin™)
Cereal		Linear β1,3/β1,4-glucan (i.e oat, barley, rye)

Figure 1. Structure of cereal and yeast Beta-glucan and scheme of various Beta-glucan sources. Adapted from Volman et al. [6].

In vitro studies

Beta-glucans enhance the functional activity of human macrophages and activate antimicrobial activity of mononuclear cells and neutrophils *in vitro* [4, 5, 12]. This enhanced immune response is accomplished by increases in pro-inflammatory cytokine and chemokine production [13-18] and oxidative burst [5, 19]. Moreover, besides leukocytes, also epithelial cells can respond to Beta-Glucans [20].

Animal studies

Next to *in-vitro* work, animal studies have been conducted to evaluate the immunomodulatory effects of Beta-glucans. Several studies have been carried out with isolated leukocytes from animals that were treated with Beta-glucans supplied via various routes. Subsequently, these isolated cells were challenged with LPS or pathogens (*ex vivo*). In general, *ex vivo* stimulated leukocytes from Beta-glucan-treated animals showed increases in pro-inflammatory cytokine production [21], oxidative burst [22], and chemotaxis [23]. Furthermore, *in vivo* studies in mice and rats have shown that intravenous or intraperitoneal Beta-glucan administration improves survival compared with control animals after challenge with fungal (Candida albicans), gram-positive (Staphylococcus. aureus), and gram-negative (Escherichia coli) pathogens [24-26].

Human in vivo evidence

Although the ability of Beta-glucan to enhance host resistance in rodents is well established, there are very few studies to support these effects in humans *in vivo*. Two clinical studies reported that intravenously administered Beta-glucan increased CD4 levels of HIV positive patients with low CD4 levels [27, 28]. It has also been reported that intravenously administered *S. cerevisiae*-derived Beta-glucan resulted in decreased infection incidence, need

for antibiotics, shortened intensive care unit length stay, and ultimately improved survival compared to placebo in high-risk surgical patients [29-31]. Furthermore, it was recently reported that oral Beta-glucan derived from *S. cerevisiae* results in enhanced expression of surface molecules associated with monocyte activation (Fas/APO-1, CD95) in patients with advanced breast cancer [32]. However, none of these studies evaluated the effects of Beta-glucan on human leukocyte function in terms of cytokine production or possible reversal of immunoparalysis.

Routes of administration
Beta-glucan is commonly administered parentally in animals and humans, while oral administration is not frequently used. This is probably due to the fact that most studies have used a Beta-glucan derived from yeast (lentinan), which is only available for parenteral use. However, in recent years a number of reports have shown that enteral administration of Beta-glucans from different sources also results in biological effects: humoral and cellular immune responses of orally administered Beta-glucan were demonstrated in animals [21], as well as enhanced resistance to bacterial and parasitic infections [33-37]. As mentioned above, in humans it was recently reported that oral Beta-glucan derived from *S. cerevisiae* results in enhanced expression of surface molecules associated with monocyte activation in patients with advanced breast cancer [32]. Moreover, systemic administration is not preferable as Beta-glucans can cause pain, granuloma's, anaphylatoxins, higher sensitivity to endotoxins, and development of micro-embolism [4]. These side-effects can be avoided by oral administration [38]. Furthermore, oral formulations of Beta-glucan are generally less expensive than their parental counterparts.

Based on these data, oral administration of Beta-glucan appears, from an immunological point-of-view, as effective as parenteral administration, is generally less expensive, and is thought to have fewer side-effects.

Study proposal
In the present application we propose to conduct a pilot study to investigate the effects of a commonly available and orally administered Beta-glucan on the innate immune response of leukocytes in humans and on faecal microbiota.

<u>Research questions:</u>

1. Does orally administered Beta-glucan modulate the *ex vivo* responsiveness of leukocytes to various inflammatory stimuli (which represents a surrogate marker for the antimicrobial response)?
2. Is orally administered Beta-glucan detectable in the blood compartment?
3. Does orally administered Beta-glucan induce changes in phenotype or gene expression caused by mechanisms other than changes in the underlying DNA sequence in leukocytes (determined by epigenetics)?
4. Does orally administered Beta-glucan affect inflammatory transcriptional pathways in leukocytes (determined by microarray)?
5. Does administration of Beta-glucan improve the capacity of leukocytes to phagocytose and kill pathogens such as the fungus *Candida albicans*?
6. To what extend does administration of Beta-glucan affect the composition of faecal microbiota?

2 OBJECTIVES

Primary objective:
The primary objective of the study is to evaluate the systemic effects of orally administered Beta-glucan on innate immune responses of leukocytes. The effects of Beta-glucan will be determined by measuring the *ex vivo* responsiveness of leukocytes to various inflammatory stimuli as a surrogate marker of the antimicrobial response, and the pathogen phagocytosis and killing capacity of leukocytes. The primary outcome measure is the TNF-α secretion by *ex vivo* LPS-stimulated peripheral blood mononuclear cells (PBMCs).

Secondary Objective(s): There are 6 secondary objectives:

1. To determine the production of other cytokines (IL-1Ra, IL-6, IL-8, IL-10, Interferon (IFN)-γ) by leukocytes *ex vivo* stimulated with various stimuli (including LPS, peptidoglycan, *Candida albicans*).

2. To determine the penetration of orally administered Beta-glucan into the blood compartment, measured by ELISA.

3. To determine the effects of Beta-glucan on changes in phenotype and gene expression caused by mechanisms other than changes in the underlying DNA sequence (epigenetic modifications).

4. To determine the effects of Beta-glucan on transcriptional pathways (by use of microarrays) with focus on inflammatory pathways.

5. To determine the effects of Beta-glucan on the leukocyte capacity to phagocytose and kill the fungal pathogen *Candida albicans*.

6. To determine the effects of Beta-glucan on faecal microbiota.

3 STUDY DESIGN

An open-label, intervention pilot-study in 15 healthy human volunteers. In this pilot study, we will enrol 10 subjects who will take Beta-glucan daily for seven days, and 5 subjects who will serve as control group and do not have to take Beta-glucan but who will be analyzed at the same time points as the subjects in the Beta-glucan group.

4 STUDY POPULATION

4.1 Population (base):

The study population consists of 15 healthy male volunteers. Before inclusion subjects must meet all inclusion criteria and none of the exclusion criteria. Recruitment of healthy volunteers will take place by placement of posters in the medical faculty and several other faculties and locations on the campus of the Radboud University Nijmegen.

4.2 Inclusion criteria

- Written informed consent
- Age ≥18
- Healthy males

4.3 Exclusion criteria

- Subjects with a history of allergy or intolerance to Beta-glucan
- Use of any medication
- Participation in a drug trial or donation of blood 3 months prior to Beta-glucan administration
- Use of antibiotics, norit, laxatives (up till 6 months prior to inclusion), cholestyramine, acid burn inhibitors or immune suppressive agents (up till 3 months prior to inclusion), and pre- and probiotics (up till 1 month prior to inclusion).

4.4 Sample size calculation

The present study is a safety and proof-of-principle pilot study. A power calculation is not warranted, as the extent (and related clinical relevance) of modulation of the innate immune response *in vivo* by the intervention is currently unknown.

5 TREATMENT OF SUBJECTS

5.1 Investigational product/treatment

In this pilot-study we will use a Beta-glucan that is commonly available as a health food supplement derived from bakers yeast (*S. cerevisiae*) and that has been used *in vivo* in mice with strong immune modulating properties and no observed toxicity [39, 40] (Glucan #300®, produced by transferpoint, Columbia, United States). Dosage is based on the dosage recommended by the manufacturer.

5.2 Use of co-intervention

Not applicable

5.3 Escape medication

Not applicable

6 INVESTIGATIONAL MEDICINAL PRODUCT

6.1 Name and description of investigational medicinal product

Commercial available Beta-glucan derived from bakers yeast (*S. cerevisiae*): Glucan #300 produced by Biothera (Eagan, Minnesota, USA) for Transferpoint (Columbia, USA).

6.2 Summary of findings from non-clinical studies

Summary of findings from non-clinical studies are described in the introduction section.

6.3 Summary of findings from clinical studies

Summary of findings from clinical studies are described in the introduction section.

6.4 Summary of known and potential risks and benefits

Purified polysaccharidic immunomodulators distinguish themselves by very low toxicity (e.g., for mice lentinan has a LD_{50} >1600mg/kg [41]). Moreover, Beta-glucans are commonly available as health food supplements and are considered safe and non-toxic, with the classification of “GRAS” (Generally recognized as safe) by the FDA as a dietary food additive supplement [42] and by the Dutch Medicines Evaluation Board (MEB) [43] (see summary of product characteristics).

6.5 Description and justification of route of administration and dosage

Oral administration is a regular route of administration (see Summary of Product Characteristics). Dosages are based on the dosage recommended by the manufacturer.

6.6 Dosages, dosage modifications and method of administration

Oral administration is a regular route of administration (see Summary of Product Characteristics). Dosages are based on the dosage recommended by the manufacturer.

6.7 Preparation and labeling of Investigational Medicinal Product

Beta-Glucan is purchased directly from manufacturer and directly delivered to the researcher in containers containing 60 capsules. The capsules will be distributed to the subjects on the first study day in, by the researcher pre-filled, medicine boxes.

6.8 Drug accountability

The products are transported to the intensive care research unit and stored there under GMP conditions.

7 METHODS

7.1 Study parameters/endpoints

7.1.1 Main study parameter/endpoints

The primary objective of the study is to evaluate the systemic effects of orally administered Beta-glucan on innate immune responses of leukocytes. The effects of Beta-glucan will be determined by measuring the *ex-vivo* responsiveness of leukocytes to various inflammatory stimuli as a marker for the antimicrobial response. The primary outcome measure is the TNF-α secretion by *ex vivo* LPS-stimulated peripheral blood mononuclear cells (PBMC's).

7.1.2 Secondary study parameters/endpoints

- production of other cytokines (TNF-, αIL-6, IL-10, IL-1β, IL-17, IL-22, Interferon (IFN)-γ) by leukocytes *ex vivo* stimulated with various stimuli (including LPS, Pam3Cys, Mycobacterium tuberculosis, Poly(I:C), Candida albicans, staphylococcus aureus)
- the absorbance of orally administered Beta-glucan into the blood compartment, measured by ELISA.
- transcriptional pathways (by use of microarrays) with focus on inflammatory pathways.
- changes in phenotype and gene expression caused by mechanisms other than changes in the underlying DNA sequence (epigenetic modifications)
- the leukocyte capacity to phagocytose and kill the fungal pathogen *Candida albicans* (antifungal activity).
- composition of faecal microbiota

7.1.3 Other study parameters

Not applicable

7.2 Randomisation, blinding and treatment allocation

We will use a open-label design in which subjects will be randomized to the Beta-glucan or control group by the opening of sealed envelopes.

7.3 Study procedures

Healthy volunteers who meet all inclusion criteria, none of the exclusion criteria and have given informed consent to participate in the study will be randomized to the Beta-glucan or the control group. The subjects in the Beta-glucan group will take Beta-glucan 1000 mg daily for 7 days, the subjects in the control group won't have to take Beta-glucan but will otherwise follow the same timecourse as the Beta-glucan treated subjects. Before, and 3, 6, and 24 hours after the first Beta-glucan ingestion (day 0), before, and 3 and 6 hours after the 7th ingestion of Beta-glucan (day 6), and on day 21 subjects have to come to the research room of the intensive care research unit for blood sampling (see appendix 1 for flowchart). On the first day of Beta-glucan ingestion we want to determine the immediate effects of oral Beta-glucan on leukocyte function, and if possible establish a plasma concentration – response curve. After 7 days of Beta-glucan ingestion we want to do the same measurements to determine

whether there is an additive effect of repetitive Beta-glucan intake. After 3 weeks we want to determine whether short-term intake of Beta-glucan results in prolonged immunological effects. Before the first intake of Beta-glucan subjects have to hand in 2 faecal samples collected within 48 hours prior to Beta-glucan intake, and one faecal sample collected within 24 hours prior to days 6 and 21. Throughout the studyperiod, subjects will document their consumption of Beta-glucan containing foods in an "eat-diary" (Appendix 2), with a maximalization of the amount of consumed Beta-glucan containing foods in order to minimize the inter-variability caused by variation in diet (Appendix 2).

7.3.1 Time course

Baseline Evaluation (day 0):

- 2 faecal samples collected within 48 hours prior to Beta-glucan intake for analysis of microbiota. Blood sampling prior to Beta-glucan intake for immunological analysis (cytokine production of leukocytes stimulated *ex vivo* with various stimuli), for analysis of peripheral blood mRNA expression of genes related to the immune response, epigenetic modifications, levels of Beta-glucan, and phagocytosis and killing of *C. albicans*.

3 hours after the first Beta-glucan intake (day 0 + 3 hours):

- Blood sampling for immunological analysis (cytokine production of leukocytes stimulated *ex vivo* with various stimuli), for analysis of peripheral blood mRNA expression of genes related to the immune response, and levels of Beta-glucan.

6 hours after the first Beta-glucan intake (day 0 + 6 hours):

- Blood sampling for immunological analysis (cytokine production of leukocytes stimulated *ex vivo* with various stimuli), for analysis of peripheral blood mRNA expression of genes related to the immune response and levels of Beta-glucan.

24 hours after the first Beta-glucan intake (day 1):

- Blood sampling for immunological analysis (cytokine production of leukocytes stimulated *ex vivo* with various stimuli), for analysis of peripheral blood mRNA expression of genes related to the immune response and levels of Beta-glucan.

Days 0-5:

- Daily intake of 1000mg Beta-glucan

Day 6:

- 1 faecal sample collected within 24 hours prior to the last Beta-glucan intake for analysis of microbiota. Blood sampling prior to the last Beta-glucan intake for immunological analysis (cytokine production of leukocytes stimulated *ex vivo* with various stimuli), for analysis of peripheral blood mRNA expression of genes related to the immune response, epigenetic modifications, levels of Beta-glucan, and phagocytosis and killing of *C. albicans*.
- Registration of any adverse experience. This includes any intercurrent illness that may arise during therapy.

3 hours after the last Beta-glucan intake (day 6 + 3 hours):

- Blood sampling for immunological analysis (cytokine production of leukocytes stimulated *ex vivo* with various stimuli), for analysis of peripheral blood mRNA expression of genes related to the immune response, levels of Beta-glucan.

6 hours after the last Beta-glucan intake (day 6 + 6 hours):

- Blood sampling for immunological analysis (cytokine production of leukocytes stimulated *ex vivo* with various stimuli), for analysis of peripheral blood mRNA expression of genes related to the immune response and levels of Beta-glucan.

Day 21:

- 1 faecal sample collected within 24 hours prior to the last Beta-glucan intake for analysis of microbiota. Blood sampling for immunological analysis (cytokine production of leukocytes stimulated *ex vivo* with various stimuli (including LPS, peptidoglycan, candida), and for analysis of peripheral blood mRNA expression of genes related to the immune response, epigenetic modifications, and levels of Beta-glucan.
- Registration of any adverse effects. This includes any intercurrent illness that may arise during the study period.

7.3.2 Laboratory tests

Immunological sampling

- Time-points: days 0, 0+3hrs, 0+6hrs, 6, 6+3hrs, 6+6hrs, and 21
- Parameters to be tested:
    - Ex-vivo cytokine production;
        - After 24h stimulation, determination of TNF-α, IFN-1, IL-6, IL-1β.
        - After 48h stimulation, determination of IL-10, IFN-γ
        - After 7 day stimulation, determination of IL-17 and IL-22
        - Method: PBMC and monocyte stimulation. Stimuli to be used: RPMI (control stimuli), LPS, Pam3Cys, Poly(I:C), *C.* albicans, staphylococcus aureus, and mycobacterium tuberculosis.
    - Peripheral blood mRNA expression of genes related to the immune response
    - Peripheral blood levels of Beta-glucan
    - Epigenetic modifications
    - phagocytosis and killing of *C. Albicans*
    - Faecal microbiota

- Total amount of blood needed: 360ml (45 ml on 8 different timepoints)
    - 40 ml EDTA-anticoagulated blood for stimulation experiments, phagocytosis/killing, and epigenetic analysis.
    - 2.5 ml collected directly into a PAXGene® Blood RNA tubes (PreAnalytix). These tubes are used to prevent any post-sampling stimulation and to ensure RNA integrity after storage. The reagents used in these BD VacutainerTM tubes allow immediate nucleic acid precipitation and nuclease inhibition. After collection and RNA stabilization at room temperature (4 hours), samples are stored at −20ºC until RNA extraction.
    - 2 ml heparin anticoagulated blood for determination of Beta-glucan by ELISA

In order to take into account the value of the biological samples obtained during this study, all leftovers will be stored in a biobank at the end of the study. This includes (if available) whole

blood, serum, plasma, RNA for the duration of 5 years. Those samples will be secondarily used for research in the field of sepsis or related pathologies. Each participant in the study will be appropriately informed and will have the right to refuse the storage of his/her own samples.

7.3.3 Methods of determination

All blood sampling will be performed on blood obtained by venapuncture. Approximately 8 x 45 ml blood will be obtained (before, and 3, 6, and 24 hours after the first Beta-glucan ingestion (day 0), before, and 3 and 6 hours after the 7th ingestion of Beta-glucan (day 6), and on day 21), with a maximum of 360 ml throughout the whole study (see appendix 1 for flowchart).

Ex vivo leukocyte stimulation will be performed at the Laboratory of Experimental Internal Medicine of the Radboud University Nijmegen Medical Centre. Cytokines in supernatants of stimulated leukocyte cultures will be determined by ELISA. At this laboratory ELISA for determination of Beta-Glucan will be performed as well.

Transcriptional activity of leukocytes will be determined by RNA sequencing at the Department of Genetics of the Radboud University Nijmegen Medical Centre. Transcriptomics analysis, systems biology, and microbiota analysis will be performed in collaboration with the group of Prof. Ramnik Xavier, Harvard University and Broad Institute at MIT.

Changes in phenotype or gene expression caused by mechanisms other than changes in the underlying DNA sequence (epigenetics) will be determined by ChIP assays at the Laboratory of Experimental Internal Medicine of the Radboud University Nijmegen Medical Centre in cooperation with the Department of Molecular Biology of the Nijmegen Centre for Molecular Life Sciences.

Analysis of phagocytosis and killing will be determined by a phagocytosis and killing assay performed at the Laboratory of Experimental Internal Medicine of the Radboud University Nijmegen Medical Centre.

7.4 Withdrawal of individual subjects

Subjects can withdraw from the study at any time without any consequences and without the need to give an explanation if they wish to do so. The investigator can decide to withdraw a subject from the study for medical reasons. Subjects that withdraw during the study will receive a proportional fee.

7.4.1 Specific criteria for withdrawal

When the subject has given informed consent, the subject is irrevocably admitted to the trial. Even if the subject is withdrawn from receiving further medication, documentation according to the study protocol must be as complete as possible.

A patient can or will be withdrawn from the study:

- upon request of the subject
- after protocol violation
- at the discretion of the investigator

7.5 Replacement of individual subjects after withdrawal

After withdrawal or exclusion of a subject, he will be replaced to maintain adequate power of the study. Maximum 10 subjects will be replaced.

7.6 Follow-up of subjects withdrawn from treatment

For all subjects who are prematurely withdrawn from treatment, the reason will be documented carefully. The patients who had a dose of Beta-glucan will at least be included in the safety evaluation. For all subjects who were withdrawn after randomization, but before Beta-glucan administration, the reason why will be documented.

7.7 Premature termination of the study

The coordinating investigators have the right to discontinue the clinical study at any time for medical or procedural reasons.

SAFETY REPORTING

7.8 Section 10 WMO event

In accordance to section 10, subsection 1, of the WMO, the investigator will inform the subjects and the reviewing accredited METC if anything occurs, on the basis of which it appears that the disadvantages of participation may be significantly greater than was foreseen in the research proposal. The study will be suspended pending further review by the accredited METC, except insofar as suspension would jeopardise the subjects' health. The investigator will take care that all subjects are kept informed.

7.9 Adverse and serious adverse events

7.9.1 Adverse event

Adverse events are defined as any undesirable experience occurring to a subject during a clinical trial, whether or not considered related to the investigational drug. All adverse events reported spontaneously by the subject or observed by the investigator or his staff will be recorded.

7.9.2 Serious Adverse Events

A serious adverse event is any untoward medical occurrence or effect that at any dose:
- results in death;
- is life threatening (at the time of the event);
- requires hospitalisation or prolongation of existing inpatients' hospitalisation;
- results in persistent or significant disability or incapacity;
- is a congenital anomaly or birth defect;
- is a new event of the trial likely to affect the safety of the subjects, such as an unexpected outcome of an adverse reaction, lack of efficacy of an IMP used for the treatment of a life threatening disease, major safety finding from a newly completed animal study, etc.

All SAEs will be reported to the accredited METC that approved the protocol, according to the requirements of that METC.

7.9.3 Suspected unexpected serious adverse reactions (SUSAR)

Adverse reactions are all untoward and unintended responses to an investigational product related to any dose administered.

Unexpected adverse reactions are adverse reactions, of which the nature, or severity, is not consistent with the applicable product information (e.g. Investigator's Brochure for an unapproved IMP or Summary of Product Characteristics (SPC) for an authorised medicinal product).

The sponsor will report expedited the following SUSARs to the METC:
- SUSARs that have arisen in the clinical trial that was assessed by the METC;
- SUSARs that have arisen in other clinical trial of the same sponsor and with the same medicinal product, and that could have consequences for the safety of the subjects involved in the clinical trial that was assessed by the METC.

The remaining SUSARs are recorded in an overview list (line-listing) that will be submitted to the METC. This line-listing provides an overview of all SUSARs from the study medicine, accompanied by a brief report highlighting the main points of concern.

The sponsor will report expedited all SUSARs to the competent authority, the Medicine Evaluation Board and the competent authorities in The Netherlands.

The expedited reporting will occur not later than 15 days after the sponsor has first knowledge of the adverse reactions. For fatal or life threatening cases the term will be maximal 7 days for a preliminary report with another 8 days for completion of the report.

7.10 Follow-up of adverse events

All adverse events will be followed until they have abated, or until a stable situation has been reached. Depending on the event, follow up may require additional tests or medical procedures as indicated, and/or referral to the general physician or a medical specialist.

7.11 Data Safety Monitoring Board (DSMB)

A DSMB is established for this study to perform ongoing safety surveillance and to perform interim analyses on the safety data. the DSMB is an independent committee completely unblinded for treatment allocation and is composed of the following persons:

Prof. dr. G.J. Scheffer, anaesthesist
Radboud University Nijmegen Medical Centre
Department of Anesthesia, PO box 9101, 6500 HB Nijmegen, The Netherlands

Prof. dr. G. Rongen, internist-pharmacologist
Radboud University Nijmegen Medical Centre
Department of Pharmacology & Toxicology, PO box 9101, 6500 HB Nijmegen, The Netherlands

One interim analysis will be performed after 7 subjects have completed the study. A DSMB-charter is attached (ATTACHMENT 1). The interim analysis will focus on the following issues: adverse events: type, severity, duration, action taken and attributability to Beta-glucan intake.

The following criteria are defined on which basis the DSMB may decide to terminate the trial prematurely:
- Any serious adverse Event

7.12 Abnormal laboratory tests results

The results of all laboratory tests required by the protocol will be recorded in the subjects case record form. All clinically important abnormal laboratory tests occurring during the study will be repeated at appropriate intervals until they return either to baseline or to a level deemed acceptable by the investigator, or until a diagnosis that explains them is made.

8 STATISTICAL ANALYSIS

8.1 Modified intention to treat analysis

Not applicable.

8.2 Descriptive statistics

For the descriptive statistics, values will be given as mean ± SD or median and ranges, depending on their distribution. For comparisons, paired Students t -tests or Willcoxon-tests for parametric and nonparametric data as appropriate will be used. Changes over time will be analysed using ANOVA repeated measures. A p value <0.05 is considered significant. Statistical analyses will be performed with SPSS 16.0 (SPSS, Chicago, IL) software.

8.3 Multivariate analysis

Not applicable.

8.4 Interim analysis

Not applicable

9 ETHICAL CONSIDERATIONS

9.1 Regulation statement

The study will be conducted according to the principles of the Declaration of Helsinki and in accordance with the Medical Research Involving Human Subjects Act (WMO).

9.2 Recruitment and consent

Subjects will be recruited by posters. They will be informed about the study and asked for their consent by the investigator. Subjects will be given at least one day to consider their decision.

9.3 Benefits and risks assessment, group relatedness

The subjects will not benefit directly from participation to the study. A subject fee is provided.

Blood withdrawal
In total, maximum 360 ml of blood will be drawn, which is not expected to result in side effects. All blood sampling will be performed on blood obtained by venapuncture.

Beta-Glucan
Beta-glucans are commonly available as health food supplements and are considered safe and non-toxic, with the classification of “GRAS” (Generally recognized as safe) by the FDA as a dietary food additive supplement [42] and by the Dutch Medicines Evaluation Board (MEB) [43].

9.4 Compensation for injury

The sponsor/investigator has a liability insurance which is in accordance with article 7, subsection 6 of the WMO.

The sponsor has an insurance which is in accordance with the legal requirements in the Netherlands (Article 7 WMO and the Measure regarding Compulsory Insurance for Clinical Research in Humans of 23th June 2003). This insurance provides cover for damage to research subjects through injury or death caused by the study.
1. € 450.000,-- (i.e. four hundred and fifty thousand Euro) for death or injury for each subject who participates in the Research;
2. € 3.500.000,-- (i.e. three million five hundred thousand Euro) for death or injury for all subjects who participate in the Research;
3. € 5.000.000,-- (i.e. five million Euro) for the total damage incurred by the organisation for all damage disclosed by scientific research for the Sponsor as ‘verrichter’ in the meaning of said Act in each year of insurance coverage.

The insurance applies to the damage that becomes apparent during the study or within 4 years after the end of the study.

9.5 Incentives

Subjects will be compensated with 200 Euro after completion of the study. If the subject ends participation before completion of the study the subject will receive a proportional share of the total fee. When the subject is forced to stop because of medical reason before the end of the study, the complete amount will be paid.

10. ADMINISTRATIVE ASPECTS, MONITORING AND PUBLICATION

10.1 Handling and storage of data and documents

Data will be handled confidentially and anonymously. A subject identification code list will be used to link the data to the subject. The code is not based on the subject initials and birth-date. The code used will be BG01.XX, where XX is the number of the subject based on the order of the screening visit (first screened subject is BG01.01 etc.).

The key to the code will be safeguarded by the principal investigator.

The handling of subject data in this study complies with the Dutch Personal Data Protection Act (in Dutch: De Wet Bescherming Persoonsgegevens, WBP).

10.2 Monitoring and Quality Assurance

Monitoring will be carried out by an internal monitor of the Radboud University Nijmegen Medical Centre after completion of the study. The stored data in the CRFs, all informed consents, SAE reports and the trial master file will be monitored.

10.3 Amendments

Not applicable.

10.4 Annual progress report

The principal investigator will submit a summary of the progress of the trial to the accredited METC once a year. Information will be provided on the date of inclusion of the first subject, numbers of subjects included and numbers of subjects that have completed the trial, serious adverse events/ serious adverse reactions, other problems, and amendments.

10.5 End of study report

The principal investigator will notify the accredited METC and the competent authority of the end of the study within a period of 90 days. The end of the study is defined as the last patient’s last visit.

In case the study is ended prematurely, the sponsor will notify the accredited METC and the competent authority within 15 days, including the reasons for the premature termination.

Within one year after the end of the study, the principal investigator will submit a final study report with the results of the study, including any publications/abstracts of the study, to the accredited METC and the Competent Authority.

10.6 Public disclosure and publication policy

The principal investigator and the coordinating investigator are first and last author of the manuscript. This trial is not sponsored by a pharmaceutical company. Authors have no conflict of interest to declare. The study will be published regardless the results of the trial.

11. STRUCTURED RISK ANALYSIS

11.1 Potential issues of concern

The subjects will not benefit directly from participation to the study. A subject fee is provided.

Blood withdrawal
Maximum 360 ml blood will be drawn, which is not expected to result in side effects. All blood sampling will be performed on blood obtained by venapuncture.

Beta-glucan
Beta-glucans are commonly available as health food supplements and are considered safe and non-toxic, with the classification of "GRAS" (Generally recognized as safe) by the FDA as a dietary food additive supplement [42] and by the Dutch Medicines Evaluation Board (MEB) [43].

12.2 Synthesis

At the end of this study, we will have assessed in humans the potential of Beta-glucan as an immune stimulatory compound. This will pave the way towards novel approaches to immunostimulatory therapies in general, and the adjunctive treatment of sepsis in particular. Because oral Beta-glucan is considered safe and non-toxic by the FDA and MEB with no documentation present of any (severe or non-severe) side effects, we feel that the remaining risks for participation in this study are negligible and do not outweigh the scientific and medical relevance of this study.

12 REFERENCES

1. Brown, G. D. and Gordon, S. (2003) Fungal beta-glucans and mammalian immunity. Immunity 19, 311-5.
2. Palma, A. S., Feizi, T., Zhang, Y., Stoll, M. S., Lawson, A. M., Diaz-Rodriguez, E., Campanero-Rhodes, M. A., Costa, J., Gordon, S., Brown, G. D., Chai, W. (2006) Ligands for the beta-glucan receptor, Dectin-1, assigned using "designer" microarrays of oligosaccharide probes (neoglycolipids) generated from glucan polysaccharides. J Biol Chem 281, 5771-9.
3. Bohn, J. A. and BeMiller, J. N. (1995) (1->3)-beta-D-glucans as biological response modifiers: A review of structure-functional activity relationships. Carbohyd Polym 28, 3-14.
4. Zekovic, D. B., Kwiatkowski, S., Vrvic, M. M., Jakovljevic, D., Moran, C. A. (2005) Natural and modified (1-->3)-beta-D-glucans in health promotion and disease alleviation. Crit Rev Biotechnol 25, 205-30.
5. Williams, D. L. (1997) Overview of (1-->3)-beta-D-glucan immunobiology. Mediators Inflamm 6, 247-50.
6. Volman, J. J., Ramakers, J. D., Plat, J. (2008) Dietary modulation of immune function by beta-glucans. Physiol Behav 94, 276-84.
7. Janeway, C. A., Jr. (1992) The immune system evolved to discriminate infectious nonself from noninfectious self. Immunol Today 13, 11-6.
8. Thornton, B. P., Vetvicka, V., Pitman, M., Goldman, R. C., Ross, G. D. (1996) Analysis of the sugar specificity and molecular location of the beta-glucan-binding lectin site of complement receptor type 3 (CD11b/CD18). J Immunol 156, 1235-46.
9. Rice, P. J., Kelley, J. L., Kogan, G., Ensley, H. E., Kalbfleisch, J. H., Browder, I. W., Williams, D. L. (2002) Human monocyte scavenger receptors are pattern recognition receptors for (1-->3)-beta-D-glucans. J Leukoc Biol 72, 140-6.
10. Zimmerman, J. W., Lindermuth, J., Fish, P. A., Palace, G. P., Stevenson, T. T., DeMong, D. E. (1998) A novel carbohydrate-glycosphingolipid interaction between a beta-(1-3)-glucan immunomodulator, PGG-glucan, and lactosylceramide of human leukocytes. J Biol Chem 273, 22014-20.
11. Brown, G. D. and Gordon, S. (2001) Immune recognition. A new receptor for beta-glucans. Nature 413, 36-7.
12. Tzianabos, A. O. (2000) Polysaccharide immunomodulators as therapeutic agents: structural aspects and biologic function. Clin Microbiol Rev 13, 523-33.
13. Engstad, C. S., Engstad, R. E., Olsen, J. O., Osterud, B. (2002) The effect of soluble beta-1,3-glucan and lipopolysaccharide on cytokine production and coagulation activation in whole blood. Int Immunopharmacol 2, 1585-97.
14. Olson, E. J., Standing, J. E., Griego-Harper, N., Hoffman, O. A., Limper, A. H. (1996) Fungal beta-glucan interacts with vitronectin and stimulates tumor necrosis factor alpha release from macrophages. Infect Immun 64, 3548-54.
15. Young, S. H., Ye, J., Frazer, D. G., Shi, X., Castranova, V. (2001) Molecular mechanism of tumor necrosis factor-alpha production in 1-->3-beta-glucan (zymosan)-activated macrophages. J Biol Chem 276, 20781-7.
16. Adachi, Y., Okazaki, M., Ohno, N., Yadomae, T. (1994) Enhancement of cytokine production by macrophages stimulated with (1-->3)-beta-D-glucan, grifolan (GRN), isolated from Grifola frondosa. Biol Pharm Bull 17, 1554-60.

17. Brown, G. D., Herre, J., Williams, D. L., Willment, J. A., Marshall, A. S., Gordon, S. (2003) Dectin-1 mediates the biological effects of beta-glucans. J Exp Med 197, 1119-24.
18. Abel, G. and Czop, J. K. (1992) Stimulation of human monocyte beta-glucan receptors by glucan particles induces production of TNF-alpha and IL-1 beta. Int J Immunopharmacol 14, 1363-73.
19. Wakshull, E., Brunke-Reese, D., Lindermuth, J., Fisette, L., Nathans, R. S., Crowley, J. J., Tufts, J. C., Zimmerman, J., Mackin, W., Adams, D. S. (1999) PGG-glucan, a soluble beta-(1,3)-glucan, enhances the oxidative burst response, microbicidal activity, and activates an NF-kappa B-like factor in human PMN: evidence for a glycosphingolipid beta-(1,3)-glucan receptor. Immunopharmacology 41, 89-107.
20. Hahn, P. Y., Evans, S. E., Kottom, T. J., Standing, J. E., Pagano, R. E., Limper, A. H. (2003) Pneumocystis carinii cell wall beta-glucan induces release of macrophage inflammatory protein-2 from alveolar epithelial cells via a lactosylceramide-mediated mechanism. J Biol Chem 278, 2043-50.
21. Vetvicka, V., Dvorak, B., Vetvickova, J., Richter, J., Krizan, J., Sima, P., Yvin, J. C. (2007) Orally administered marine (1-->3)-beta-D-glucan Phycarine stimulates both humoral and cellular immunity. Int J Biol Macromol 40, 291-8.
22. Mucksova, J., Babicek, K., Pospisil, M. (2001) Particulate 1,3-beta-D-glucan, carboxymethylglucan and sulfoethylglucan--influence of their oral or intraperitoneal administration on immunological respondence of mice. Folia Microbiol (Praha) 46, 559-63.
23. LeBlanc, B. W., Albina, J. E., Reichner, J. S. (2006) The effect of PGG-beta-glucan on neutrophil chemotaxis in vivo. J Leukoc Biol 79, 667-75.
24. Liang, J., Melican, D., Cafro, L., Palace, G., Fisette, L., Armstrong, R., Patchen, M. L. (1998) Enhanced clearance of a multiple antibiotic resistant Staphylococcus aureus in rats treated with PGG-glucan is associated with increased leukocyte counts and increased neutrophil oxidative burst activity. Int J Immunopharmacol 20, 595-614.
25. Lagrange, P. H., Fourgeaud, M., Neway, T., Pilet, C. (1994) Enhanced resistance against lethal disseminated Candida albicans infection in mice treated with polar glycopeptidolipids from Mycobacterium chelonae (pGPL-Mc). C R Acad Sci III 317, 1107-13.
26. Onderdonk, A. B., Cisneros, R. L., Hinkson, P., Ostroff, G. (1992) Anti-infective effect of poly-beta 1-6-glucotriosyl-beta 1-3-glucopyranose glucan in vivo. Infect Immun 60, 1642-7.
27. Gordon, M., Guralnik, M., Kaneko, Y., Mimura, T., Goodgame, J., DeMarzo, C., Pierce, D., Baker, M., Lang, W. (1995) A phase II controlled study of a combination of the immune modulator, lentinan, with didanosine (ddI) in HIV patients with CD4 cells of 200-500/mm3. J Med 26, 193-207.
28. Gordon, M., Bihari, B., Goosby, E., Gorter, R., Greco, M., Guralnik, M., Mimura, T., Rudinicki, V., Wong, R., Kaneko, Y. (1998) A placebo-controlled trial of the immune modulator, lentinan, in HIV-positive patients: a phase I/II trial. J Med 29, 305-30.
29. Babineau, T. J., Hackford, A., Kenler, A., Bistrian, B., Forse, R. A., Fairchild, P. G., Heard, S., Keroack, M., Caushaj, P., Benotti, P. (1994) A phase II multicenter, double-blind, randomized, placebo-controlled study of three dosages of an immunomodulator (PGG-glucan) in high-risk surgical patients. Arch Surg 129, 1204-10.
30. Babineau, T. J., Marcello, P., Swails, W., Kenler, A., Bistrian, B., Forse, R. A. (1994) Randomized phase I/II trial of a macrophage-specific immunomodulator (PGG-glucan) in high-risk surgical patients. Ann Surg 220, 601-9.

31. Dellinger, E. P., Babineau, T. J., Bleicher, P., Kaiser, A. B., Seibert, G. B., Postier, R. G., Vogel, S. B., Norman, J., Kaufman, D., Galandiuk, S., Condon, R. E. (1999) Effect of PGG-glucan on the rate of serious postoperative infection or death observed after high-risk gastrointestinal operations. Betafectin Gastrointestinal Study Group. Arch Surg 134, 977-83.
32. Demir, G., Klein, H. O., Mandel-Molinas, N., Tuzuner, N. (2007) Beta glucan induces proliferation and activation of monocytes in peripheral blood of patients with advanced breast cancer. Int Immunopharmacol 7, 113-6.
33. Yun, C. H., Estrada, A., Van Kessel, A., Gajadhar, A. A., Redmond, M. J., Laarveld, B. (1997) beta-(1-->3, 1-->4) oat glucan enhances resistance to Eimeria vermiformis infection in immunosuppressed mice. Int J Parasitol 27, 329-37.
34. Kournikakis, B., Mandeville, R., Brousseau, P., Ostroff, G. (2003) Anthrax-protective effects of yeast beta 1,3 glucans. MedGenMed 5, 1.
35. Lowry, V. K., Farnell, M. B., Ferro, P. J., Swaggerty, C. L., Bahl, A., Kogut, M. H. (2005) Purified beta-glucan as an abiotic feed additive up-regulates the innate immune response in immature chickens against Salmonella enterica serovar Enteritidis. Int J Food Microbiol 98, 309-18.
36. Yun, C. H., Estrada, A., Van Kessel, A., Park, B. C., Laarveld, B. (2003) beta-glucan, extracted from oat, enhances disease resistance against bacterial and parasitic infections. Fems Immunol Med Mic 35, 67-75.
37. Yun, C. H., Estrada, A., Van Kessel, A., Gajadhar, A., Redmond, M., Laarveld, B. (1998) Immunomodulatory effects of oat beta-glucan administered intragastrically or parenterally on mice infected with Eimeria vermiformis. Microbiology and Immunology 42, 457-465.
38. Hunter, K. W., Jr., Gault, R. A., Berner, M. D. (2002) Preparation of microparticulate beta-glucan from Saccharomyces cerevisiae for use in immune potentiation. Lett Appl Microbiol 35, 267-71.
39. Vetvicka, V. and Vetvickova, J. (2007) Physiological effects of different types of beta-glucan. Biomed Pap Med Fac Univ Palacky Olomouc Czech Repub 151, 225-31.
40. Vetvicka, V. and Vancikova, Z. (2010) Anti-stress action of several orally-given beta-glucans. Biomed Pap Med Fac Univ Palacky Olomouc Czech Repub 154, 235-8.
41. Chihara, G., Maeda, Y. Y., Hamuro, J. (1982) Current status and perspectives of immunomodulators of microbial origin. Int J Tissue React 4, 207-25.
42. FDA http://www.fda.gov/Food/FoodIngredientsPackaging/GenerallyRecognizedasSafeGRAS/GRASListings/ucm153925.htm.
43. MEB http://www.cbg-meb.nl/CBG/en/novel-foods/definition-novel-foods/Yeast_beta-glucans/default.htm.

APPENDIX 1 Flowchart Beta-glucan pilot study

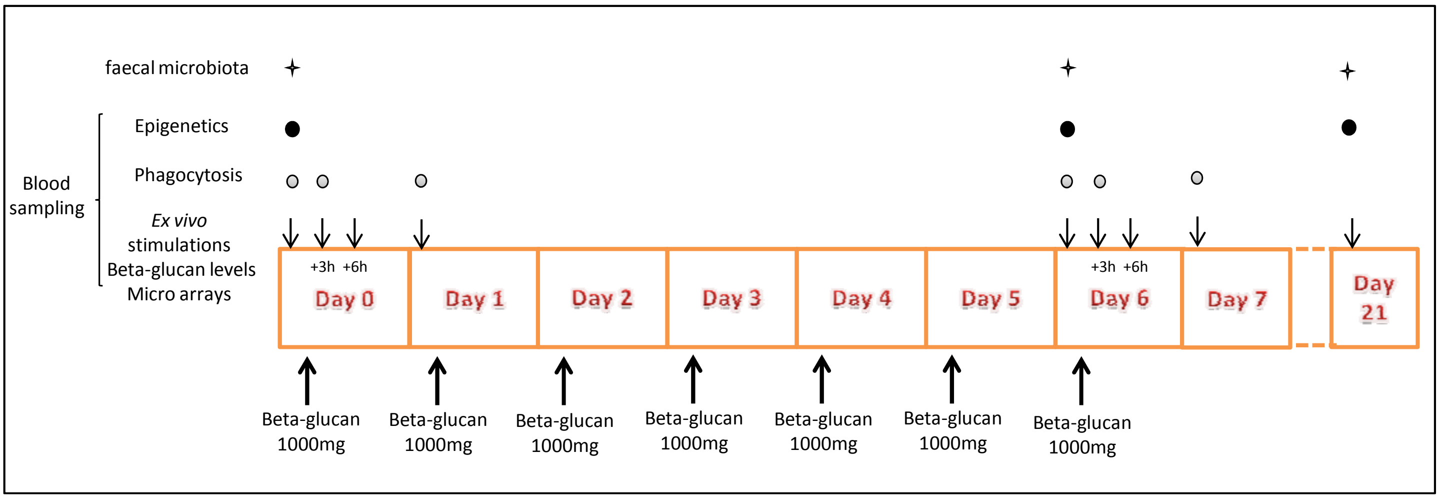

Supplement: Protocol S1 — Protocol of this trial. (PDF) [file pone.0108794.s003.pdf]
